# Revisiting Anterior Cruciate Ligament Repairs in an Athlete With Combined Grade III Medial Collateral Ligament and High-Grade Posterolateral Anterior Cruciate Ligament Tear: A Case Report

**DOI:** 10.7759/cureus.49522

**Published:** 2023-11-27

**Authors:** Sean M Muir, Alyssa McMandon, Emily Sadowski, John Lucas, James D McDermott

**Affiliations:** 1 Medicine, Edward Via College of Osteopathic Medicine, Spartanburg, USA; 2 Surgery, Edward Via College of Osteopathic Medicine, Spartanburg, USA; 3 Sports Medicine, Spartanburg Medical Center, Spartanburg, USA; 4 Orthopedic Surgery, Spartanburg Medical Center, Spartanburg, USA

**Keywords:** athlete, medial collateral ligament repair, anterior cruciate ligament repair, medial collateral ligament injury, anterior cruciate ligament injury, knee

## Abstract

Anterior cruciate ligament tears are primarily treated by reconstruction. The development of novel surgical techniques has led to the reconsideration of this approach. Additionally, Grade III tibial-sided medial collateral ligament tears should be treated surgically due to decreased blood flow and poor healing. We describe the surgical repair of a Grade III tibial-sided tear with partial femoral avulsion of the medial collateral ligament and tear of the posterolateral bundle of the anterior cruciate ligament in a competitive high school athlete.

A 17-year-old male presented to the Sports Medicine Clinic after injuring his left knee in a football game. Radiographs suggested normal skeletal anatomical alignment with no acute fractures. Magnetic resonance imaging identified a partial injury of the femoral attachment of the medial collateral ligament and a Grade III medial collateral ligament tear where it attached to the tibia. Arthroscopic evaluation of the knee revealed a posterolateral anterior cruciate ligament tear. Operative management included surgical repair of the Grade III tibial-sided medial collateral ligament tear and the posterolateral anterior cruciate ligament tear. Operative repair of medial collateral ligament and anterior cruciate ligament tears provides an alternative approach to the management of surgical reconstruction.

## Introduction

The most common multi-ligamentous knee injuries are tears of the medial collateral and anterior cruciate ligaments (MCL-ACL) [1]. ACL tears are commonly associated with Grade III (i.e., complete) MCL tears and are infrequently found with Grade I (<10%) MCL tears. Indications for operative repair of Grade III MCL tears include multi-ligamentous injuries, chronic instability, non-operative management failure, or distal avulsion with a Stener-like lesion where the torn end becomes entrapped in the medial compartment [1-4]. Some Grade III MCL injuries have been successfully managed non-operatively; however, most orthopedic sports medicine surgeons recommend repair to limit complications e.g., (chronic pain, instability, reinjury) associated with non-operative management. Operative repair may provide additional benefits, especially in athletes because it limits complications and shortens the time to play [5]. Notably, tibial-sided MCL tears receive less blood flow than femoral-sided tears and are predisposed to delayed or diminished healing requiring operative repair [6,7]. Contemporary recommendations suggest nonoperative management of Grade I and II MCL injuries and operative reconstruction of ACL tears when Grade III MCL tears are present [1].

Historical data suggested that the risk of reinjury following ACL repair could be as high as 20% and the procedure was abandoned [8]. ACL reconstruction is the current standard of care for MCL-ACL injuries and isolated ACL tears [9]. It involves using a patellar, quadriceps, or hamstring tendon autograph to rebuild the ACL ligament [10]. ACL repair, however, may be a viable surgical alternative in select patients. Recent research has shown that ACL repair can benefit specific femoral avulsions and isolated posterolateral bundle tears, particularly in younger patients [11,12]. This case report describes the surgical management of an MCL-ACL repair in a high school football athlete.

## Case presentation

A 17-year-old male athlete presented to the Sports Medicine Clinic after injuring his left knee in a football game. His left foot was planted when he was hit causing his knee to hyperextend. There was no “pop” noted during the incident, however, he immediately observed swelling. No endpoint with valgus stress was noted during the anterior-posterior (A-P) translation of the tibia. The radiographic evaluation suggested normal alignment without dislocation, normal joint spaces, no acute fractures, and a large amount of joint effusion. Ultrasound-guided aspiration of the left knee joint produced 30 mL of blood-colored fluid. An MRI, without contrast, of the left knee was requested and the patient was referred to orthopedic surgery.

**Figure 1:**
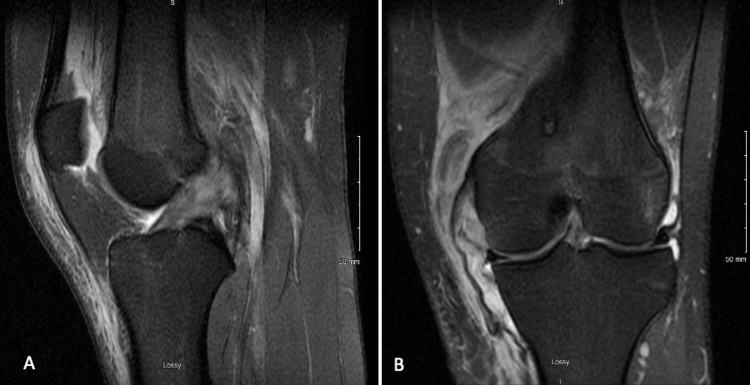
MRI demonstrating Grade III tibial-sided MCL tear (A) and an anteromedial ACL tear (B).

The MRI confirmed a Grade III MCL tear at its attachment to the tibia with partial injury of the femoral attachment. A partial tear of the ACL involving the anteromedial bundle was also noted (Figures 1A, 1B). The treatment alternatives for operative management were reviewed with the patient and the decision was made to surgically repair the Grade III MCL tear because tibial-sided tears have poorer vascular supply than femoral-sided tears and do not heal as well as femoral-sided tears [6,7]. Most ACL tears are treated by surgical reconstruction due to the high failure rates as previously noted [8]. More recently, however, isolated ACL bundle tear repairs have become popular [11,12]. The medical management options were explained to the patient, and it was agreed that if there was an isolated A-M ACL bundle tear it would be repaired, however, if reconstruction was needed a patellar-tendon allograft would be used. The patient consented to the surgical plan of diagnostic arthroscopy, tibial-sided MCL repair, and ACL repair or reconstruction.

An orthopedic ligamentous examination during anesthesia displayed a firm endpoint with an anterior drawer and a 1A Lachman’s test. A slightly positive pivot shift, a 2-3+ joint line opening to varus stress at 30 degrees of flexion, and a 1+ joint line opening at 0 degrees of extension were also noted and recorded. Diagnostic arthroscopy confirmed extensive hemarthrosis that was suctioned and cleaned. Arthroscopy of the patellofemoral, medial, and lateral compartments revealed intact cartilage. Both medial and lateral menisci were intact. Probing of the ACL revealed a posterolateral ACL tear, not an anteromedial tear as reported on the MRI. This ACL injury was likely responsible for the extensive joint capsule hemarthrosis.

**Figure 2:**
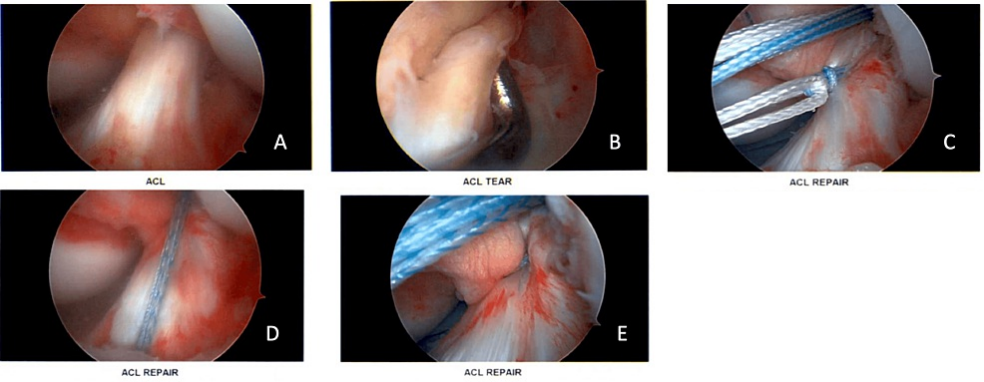
Primary ACL repair demonstrating (A) anteromedial ACL bundle. (B) Torn posterolateral ACL bundle. (C-E) ACL repair using TightRope and FiberRing.

Tunnels are created both through the femur and the tibia for threading suture material through during primary ACL repair. The femoral tunnel was made by bringing the knee into hyperflexion, a guide marks the correct anatomical position, and a reamer was used to drill through the bone. Similar steps were employed to make the tibial tunnel. Suture materials (TightRopeÒ and FiberRingÒ) were threaded through the avulsed portion of the P-L bundle and then passed through the femoral and tibial tunnels as described in the Arthrex technique guide (Figures 2A-2E) [13]. This suture material is used to re-anchor the torn portion of the ACL ligament back into the correct anatomical location. An adjustable suspensory button fixation (i.e., flip button) presses against the distal side of the tunnel and is used to lock the suture material in place. Visual inspection and assessment of the ACL repair confirmed that the suture material was not irritating the ACL during a full range of motion.

The MCL was repaired by making surgical incisions over the medial femoral condyle and medial tibial plateau followed by dissection of soft tissue to the sartorial fascia. The tibial-sided MCL tear was confirmed and the semimembranosus, gracilis, and medial attachments of the hamstrings were identified. A guide was used to drill a tunnel anterior to posterior to the MCL to pass suture material through and anchor the MCL in the bone. Suture material was passed through the MCL employing a modified Krackow technique [14]. Once the footprint of the MCL was restored the sutures were passed through each tunnel. The knee was placed at 30 degrees in neutral rotation and the sutures were tied with half-hitch knots. Stability assessment of the knee was determined at zero- and 30-degree flexion and firm endpoints were noted with valgus stress. The wound was irrigated and closed in layers and occlusive dressings were placed on the wound site.

**Figure 3:**
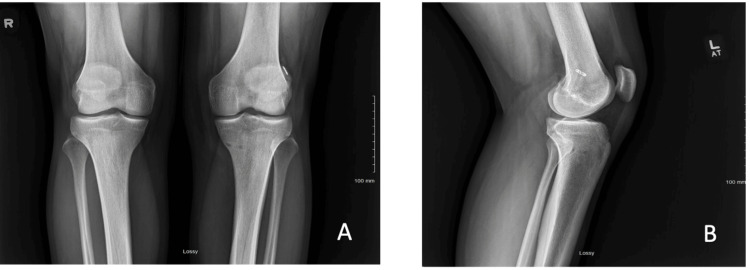
Three-month post-operative x-rays. (A) Anterior-posterior views of the knee. (B) Lateral view of the knee.

Radiographs obtained three months after surgery (Figures 3A, 3B) demonstrated a well-healed primary ACL and MCL. The incision was intact without drainage and was stable on ligamentous examination. The patient adhered to the MCL and ACL repair protocol, initiating toe-touch weight-bearing post-surgery with a locked brace in extension. From weeks 2-4, they engaged in 50% partial weightbearing, progressing to weightbearing as tolerated from weeks 4-6. Range of motion (ROM) was encouraged at 0-60 degrees from weeks 2-4 and 0-90 degrees from weeks 4-6. The brace was removed at the four-week mark, followed by the commencement of strengthening exercises in physical therapy from weeks 4-6. By the three-month mark, the patient had achieved a full passive and active range of motion, was pain-free, and demonstrated good strength. At four months, the patient initiated cutting and non-contact activities during football practice. A return-to-play (RTP) test was conducted at five months post-surgery, and clearance for full-contact football was granted at the six-month mark.

## Discussion

Grade I and II MCL injuries with or without concomitant ACL injuries are currently treated nonoperatively [1]. Some Grade III MCL injuries are treated nonoperatively although operative management is recommended in patients with severe multi-ligamentous injury, chronic instability, distal avulsion with a Stener-type entrapment of the torn end in the medial compartment, and nonoperative management failure [15]. Nonoperative treatment has been shown to increase return to playtime and reinjury and decrease field time after return to play in elite-level athletes [16] These findings have caused most sports medicine orthopedic surgeons to select operative management of Grade III MCL tears in athletes [5]. Acevedo et al. were one of the first to demonstrate the success of operative management in Grade III MCL tears with an appropriate post-operative protocol in Division I football athletes [5]. Others have demonstrated that a wave sign detected on MRI is indicative of tibial-sided MCL avulsion that requires acute surgical repair “for maximum benefit” [17].

Phisitkul et al. have reviewed the complications associated with MCL repair, the benefits of MCL repair, and the need for further studies comparing nonoperative repair to operative repair [1]. This study emphasized the need for further investigation of MCL-ACL management options. Holuba et al. demonstrated that nonoperative management of the MCL tears with an operative reconstruction of the ACL produced poor results due to an increase in valgus laxity, return to play time, and risk of intra-articular damage. The same authors also suggested that treatment algorithms for MCL-ACL injuries need to be revisited based on historical outcomes [3]. Koga et al. recently demonstrated that primary MCL repair with ACL reconstruction produced limited post-operative complications [4]. Similarly, Van der List et al. attributed the successful repair of MCL-ACL injuries with primary MCL repair and ACL reconstruction to the prevention of muscle atrophy, valgus instability, and the maintenance of proprioception [2]. ACL repair, however, is being reconsidered in select situations because of advancements in surgical techniques and new technologies [12]. For example, a one-year follow-up study has demonstrated successful functional recovery and low refracture rates in a primary ACL repair using dynamic intraligamentary stabilization [18]. In addition, DiFelice et al. have demonstrated short-term success of proximal avulsion-type tears treated with an arthroscopic primary ACL repair technique [19].

## Conclusions

Primary ACL repair can be considered in athletes with partial ACL tears. Operative management is recommended for athletes with MCL-ACL injuries especially those with tibial-sided MCL tears. Further evaluation of ACL repair documenting return to play time, risk of reinjury, and other complications is required in patients with operative and non-operative Grade III MCL repair. Primary ACL repair is a potential option for some athletes with Grade III MCL-ACL multi-ligamentous knee injuries and may provide improved outcomes compared to ACL reconstruction.
